# Hospital Administration

**Published:** 1894-12-15

**Authors:** 


### HOSPITAL ADMINISTRATION.

[Questions and correspondence, marked "Hospital Administration" on the envelope, addressed to the Editor, are invited for this column.]

### Working Men Representation.

An interesting discussion recently took place at Dudley, at the annual meeting of the Guest Hospital, on this subject, which was ultimately adjourned for six weeks, when a special meeting of Governors will be held to settle the matter. Three proposals were made: (1) That any body of working men, company, or association subscribing in their collective capacity an annual sum of not less than two guineas should appoint a representative to have one vote at the annual meeting on production of written evidence of his election; as an alternative, (2) that any clergyman or representative of a congregation, or any appointed representative of any club, society, or body of working people, or any person collecting in one year £10, so long as such payment be annually made, shall have a vote at the annual and special meetings, and be eligible for election on the committee of management; (3) that the secretary be instructed to form an outside committee to manage the working men's collections, and that the chairman of that committee be an *ex-officio* member of the Hospital Weekly Board and Committee.

Dr. Messiter contended on the one hand, in speaking against the first proposal, that it meant that the working men would have a majority of votes at the annual meeting, and that they might, by coming into conflict with the authorities, bring the whole work of the institution to a dead lock. To this it was replied that it was absurd to contend that the working men representatives would attend the meetings and the subscribers stay away; but this argument lost its force from the fact that less than thirty governors were present.

In our view it is desirable that working men should be represented on the committee of management. It is now the general practice to appoint two visitors from the working-class governors, and to elect on the committee two representatives of the Hospital Saturday Fund or Workshop Collections Fund.

### The Importance of Fever Hospitals.

By a singular coincidence, whilst the Government arbitrator was settling certain questions as to the site of the new fever hospital at Kingstown, Ireland, a poor woman, in the early stage of small-pox, came to the Town Hall to know where she was to go. There was no accommodation of any kind in Kingstown, and she had to be taken in the ambulance to the Cork Street Fever Hospital, Dublin, a journey of about ten miles. The absence of fever accommodation is not confined to

Kingstown, however, and the sooner the inhabitants of every community recognise the importance of having adequate fever accommodation within easy reach of their homes, the better it will be for themselves, their wives, and their children.

Hospital Sunday Amenities.

At a meeting of the governors of the Coventry and Warwickshire Hospital on the 26th ult., Mr. Pulley complained that the largest and richest congregation in Coventry—that of St. Michael's—had given no collection to the hospital for three years past. This statement has led the Rev. Dr. Mills, the vicar of St. Michael's, to reply that three years ago he was compelled to withdraw his annual subscription, and to refuse to include this hospital amongst the charities which he officially puts before his congregation. At the time Dr. Mills gave his reasons in writing to the committee, and within the last three months he had again put in writing his reasons, at the request of one of the officials. Dr. Mills declared that these reasons are known to the committee, and are unanswerable.

It is important that the reasons in question should be made public in the interests of the patients and of the institution concerned. Dr. Mills' remarks imply most grievous reflections upon the management of the hospital, especially as he concludes his defence with the statement that "he has fought many a hard battle for his God and his Church, and Birmingham readers will understand and appreciate his conduct in neither raking up the past sad history or permitting himself to be drawn into further correspondence." We shall be glad to hear from the authorities of the Coventry and Warwickshire Hospital without delay.

Local Government Board Inspectors and the Sick Poor.

Sir Walter Foster should look after Mr. Longe, one of the Local Government Board inspectors, whose remarks as reported in a local paper demand some explanation at his hands. The chairman of the Kidderminster Guardians having stated that they were bound to accede to the request of their medical officer to engage more nurses and to do the very best they could for the sick poor, Mr. Longe is reported to have expressed surprise that such highly paid nurses should be engaged. Mr. Longe did not profess to say "what the medical man had a right to do, but it was perfectly monstrous that such expenses as four guineas a week should be incurred for the nursing of one patient. No poor person outside the workhouse, not even tradesmen or people in higher classes, would have two such highly paid nurses." Mr. Longe ultimately admitted that much depended upon circumstances, and that typhoid fever cases were very difficult ones to deal with; still he declared the Board were clearly not justified in incurring any extraordinary expenses in these cases. The chairman, we are glad to see, pointed out that the responsibility rested upon the medical officer, who had accepted it. If the Local Government Board Inspector is to take under his control the treatment of the patients in the Poor Law infirmaries and workhouses throughout the country, it is quite certain that the patients will have a short shrift and small consideration if we may regard Mr. Longe as a typical inspector.

The Appointment of Resident Medical Officers.

It is usual for committees of management to appoint the house physicians and house surgeons. At Grimsby, however, the practice prevails of referring all the applicants to the honorary medical staff of the hospital, and if they unanimously recommend one candidate, his appointment is forthwith ratified by the committee. If there is a difference of opinion between the members of the hon. medical staff then the candidates are referred back to the committee, who proceed to elect one of them in due course. It appears to us that the Grimsby plan has much to recommend it, and we should be glad to know how far it has been tried at other hospitals.

Hospital Expenditure and the Daily Press.

We welcome the co-operation of the *Westminster Gazette* in the cause of sound hospital administration. Mr. Cook is publishing some excellent articles on hospital expenditure, moderate in tone and very useful as a contribution by the lay Press to a somewhat complicated subject. The Press all over the country has now at its command such a mass of new and accurate information every year in "Burdett's Hospital and Charities Annual," that we hope many other newspapers will take up hospital questions from time to time, and devote more space to the subject than they have ever done before. The wider the interest and the fiercer the criticism, the greater will be the support extended to the voluntary hospitals by the public at large. It follows that all the more intelligent and active managers desire to secure the widest publicity. This can only be obtained by the co-operation of the Press, to whom we commend the example set by the *Westminster Gazette* as a new departure and precedent worthy of being generally followed all over the country

